# Anticancer Activity of Urease Mimetic Cobalt (III) Complexes on A549-Lung Cancer Cells: Targeting the Acidic Microenvironment

**DOI:** 10.3390/pharmaceutics14010211

**Published:** 2022-01-17

**Authors:** Bhawna Uprety, Rahul Chandran, Charmaine Arderne, Heidi Abrahamse

**Affiliations:** 1 Laser Research Centre, Faculty of Health Sciences, University of Johannesburg, P.O. Box 17011, Johannesburg 2028, South Africa; habrahamse@uj.ac.za; 2 Research Centre for Synthesis and Catalysis, Department of Chemical Sciences, University of Johannesburg, P.O. Box 524, Johannesburg 2092, South Africa; carderne@uj.ac.za

**Keywords:** urease mimetic activity, tumour microenvironment, cobalt (III) complexes

## Abstract

Tumour cells maintain a local hypoxic and acidic microenvironment which plays a crucial role in cancer progression and drug resistance. Urease is a metallohydrolases that catalyses the hydrolysis of urea into ammonia and carbon dioxide, causing an abrupt increase of pH. This enzymatic activity can be employed to target the acidic tumour microenvironment. In this study, we present the anticancer activities of urease mimetic cobalt (III) complexes on A549 cells. The cells were treated with different doses of cobalt (III) complexes to observe the cytotoxicity. The change in cellular morphology was observed using an inverted microscope. The cell death induced by these complexes was analysed through ATP proliferation, LDH release and caspase 3/7 activity. The effect of extracellular alkalinization by the cobalt (III) complexes on the efficacy of the weakly basic drug, doxorubicin (dox) was also evaluated. This combination therapy of dox with cobalt (III) complexes resulted in enhanced apoptosis in A549 cells, as evidenced by elevated caspase 3/7 activity in treated groups. The study confirms the urease mimicking anticancer activity of cobalt (III) complexes by neutralizing the tumour microenvironment. This study will motivate the applications of transition metal-based enzyme mimics in targeting the tumour microenvironment for effective anticancer treatments.

## 1. Introduction

The last few decades have seen an upsurge in cancer research. Many developments and milestones have been achieved in discovering compounds with effective anticancer therapeutic properties. Although a lot of such potent drugs such as doxorubicin and cisplatin were discovered several years ago and are still used clinically to treat different kinds of cancers; they are often limited by severe side effects on healthy cells [1,2]. Recently, the research focus has shifted to develop target specific treatments. These involve the design of target specific drug delivery agents such as using nanotechnology [3,4,5,6], immunotherapy [7,8], and more recently, antibody conjugation [9,10,11] to allow delivery of the drug molecule directly at the tumour site. These procedures are believed to improve drug efficacy while minimizing adverse side effects [12]. Although considerable advancements have been achieved, most of these treatments often require chronic therapy and offer only moderate effects. Therefore, new treatment methods are in demand to fight this deadly disease.

Recently, some researchers have focused on targeting the microenvironment surrounding tumour cells [13,14,15,16,17]. Hypoxia plays a crucial role in suppressing the body’s immune response and proliferation of tumour cells, which tend to become heterogeneous at anatomic, genomic, and metabolic levels. This may be due to the deficiency of essential nutrients and oxygen around cancer cells [18]. Another implication of the hypoxic microenvironment is the increased generation of non-oxidized acids such as lactic acid in the tumour extracellular environment [19]. Consequently, the pH around tumour cells could be lowered to up to 6.2 [19,20]. The acidosis of the tumour extracellular environment has multi-fold effects on tumour progression; it suppresses the body’s immune response and stimulates tumour growth factors, and also interferes with the action of drug molecules by altering the formal charge on the molecule, thereby shifting the partition coefficient between the intracellular and the extracellular regions. This results in greater localization of weakly basic drugs in the extracellular region, thus decreasing the drug efficacy. Tumour cells that have adapted to a low extracellular pH also exhibit greater expression of p-glycoproteins, which ultimately results in drug resistance [21]. Therefore, current research is now focused on the alkalinization of the extracellular microenvironment to promote the efficacy of existing treatments. Some of the most promising results were obtained by Chao et al. using the urease enzyme [22].

Urease (urea amidohydrolase; E.C.3.5.1.5) is widespread in nature and is present in various forms in several bacteria, fungi, and algae [23]. It is commonly associated with the pathogenesis of various pathogens such as Helicobacter pylori and Mycobacterium tuberculosis. These bacteria survive the acidic conditions of the gut by virtue of their intrinsic urease activity to neutralize the acidity [24]. Likewise, urease activity is also responsible for the ineffective use of nitrogen fertilizers. Urease rapidly hydrolyses urea into ammonia, most of which is dissipated into the environment, rather than being absorbed by the plants [24]. Therefore, much research has been dedicated to inhibiting the enzyme activity to attain the desired therapeutic as well as agricultural benefits [23,25,26,27,28,29,30,31,32]; however, the last decade has seen potential urease mimicking applications [33,34,35,36]. The most promising results have been documented for the anticancer applications of the urease enzyme. Chao et al. have developed a series of urease conjugates (DOS47) to induce alkalinization of the interstitial medium surrounding the tumour cells by promoting the hydrolysis of urea to generate ammonia; they also reported a substantial improvement in the anticancer effect of weak base anticancer drugs (doxorubicin and vinblastine) when used synergistically with the urease enzyme [22,37,38,39]. However, the greatest challenge working with actual enzymes pertains to isolation and storage of the enzyme from different sources, making it a tedious and non-economic process. Moreover, since the enzymes are proteins and can be easily denatured by small changes in temperature and pressure, they provide a very narrow window to explore the desired results.

## 2. Materials and Methods

### 2.1. Materials-Physical Measurements

All chemicals were used as received without further purification and were of the analytical grade. CoCl_2_·6H_2_O (Sigma-Aldrich, Johannesburg, South Africa), tris(2–aminoethyl)amine (Sigma-Aldrich, Johannesburg, South Africa), triethylaminetetramine (Sigma-Aldrich, Johannesburg, South Africa), and urea (Saarchem, Johannesburg, South Africa) were used as obtained. The complexes were prepared following literature methods [33] using the respective ligands. FT–IR spectra were recorded in Attenuated total reflection mode on a Spectrum BX, Perkin–Elmer FT–IR spectrometer (Perkin–Elmer, Waltham, MA, USA). Electronic spectra of complexes were recorded in water on a Shimadzu 1800 UV–Vis spectrophotometer (Shimadzu, Kyoto, Japan). ^1^H NMR and ^13^C NMR spectra were recorded in D_2_O/CH_3_OD on a Bruker Ultrashield 400 Nuclear Magnetic Resonance Spectrometer (Bruker, Billerica, MA, USA). SC-XRD data were obtained on a Bruker Apex–II Duo CCD diffractometer (Bruker). The spectroscopic characterization data for the complexes has been included in the supplementary information.

### 2.2. Cell Culture and Treatment

Commercially purchased lung cancer (A549-ATCC CCL-185), MCF7 breast cancer cell line (ATCC HTB-22), Oesophageal cancer (HKESC-1, Cellonex-SepSci, Johannesburg, South Africa) and fibroblast (WS1-ATCC: CRL-1502′ to ATCC^®^ CRL-1502™) cell lines were used for the cytotoxicity study. Here, MCF-7 and HKESC-1 were used as additional cell lines to determine the toxic response of cobalt (III) complexes. Approximately, 3 × 10^5^ cells were seeded in 3.4 cm diameter culture dishes. A549 cells were cultured in Roswell Park Memorial Institute 1640 Medium (RPMI, Sigma-Aldrich, R8758, Johannesburg, SA, South Africa) supplemented with 10% foetal bovine serum (FBS, Gibco, 306.00301), 1% antibacterial (Penicillin-streptomycin-Sigma-Aldrich, Johannesburg, SA, South Africa) and 1% antifungal (amphotericin-B, Gibco, Thermofisher, 104813, Johannesburg, SA, South Africa) agents; however, MCF-7 and HKESC-1 cells were cultured in Dulbecco’s modified Eagle’s media (DMEM, Sigma-Aldrich, D 6429) with supplements and antibiotics, as mentioned above. WS1 cells were cultured in Minimum Essential Media (MEM-Sigma-Aldrich, Johannesburg, SA, South Africa) with 1% Amphotericin-B (Sigma-Aldrich, Johannesburg, South Africa), 1% penicillin-streptomycin (Sigma-Aldrich, P4333, Johannesburg, South Africa), 0.1 mM Non-essential amino acid (Sigma-Aldrich, M7145, Johannesburg, South Africa), 1 mM sodium pyruvate (Sigma-Aldrich, S8636, Johannesburg, South Africa), 2 mM l-glutamine (Sigma-Aldrich, G7513, Johannesburg, South Africa), and 10% foetal bovine serum (Gibco, 306.00301). The cultures were maintained at 37 °C with 5% CO_2_ and 85% humidity for 4 h to allow the cells to attach. Culture dishes with more than 90% confluence were used for these experiments. The experimental groups were divided into untreated controls and cells treated with the drugs at three different doses (8 μM, 16 μM, and 32 μM).

### 2.3. Stability of Co (III) Complexes

Since the study is focused on the toxicity of the complexes against lung cancer cells, the stability of the compounds in the A549 cell culture medium was analysed by UV/Vis spectroscopy as well as by SC-XRD analyses. A solution of all the complexes was made (~7 mM, 1 mL) by dissolving the compounds in a solution of RPMI supplemented with 10% foetal bovine serum, 1% penicillin-streptomycin and 1% amphotericin-B. The resulting solutions were maintained at 37 °C, and their UV/Vis spectra were analysed for 3 days, every 12 h.

### 2.4. Urease Activity

The urease mimetic activity of the chloro complexes, ([Co(trien)Cl_2_]Cl (**III**) and [Co(tren)Cl_2_]Cl (**IV**)) was measured from the culture media of the treated and untreated groups. 100 μL of the culture media was withdrawn from treated and untreated groups at fixed time intervals and the urease mimetic activity was measured using the urease activity kit from Merck Sigma-Aldrich, MAK120, following the protocol provided.

### 2.5. Cellular Morphology-Inverted Microscopy

The morphology of the complexes (8 μM, 16 μM, and 32 μM) treated cells was analysed after 24 h of incubation using an inverted light microscope (Wirsam, Olympus CKX41, Johannesburg, South Africa). After the digital images were recorded, cells were detached using 1 mL/plate of TrypLE Express (Invitrogen, 12605-028), centrifuged at 3000 rpm for 5 min and re-suspended in Hank’s Balanced Salt Solution (HBSS) to perform further assays.

### 2.6. Cellular Proliferation-Adenosine Triphosphate (ATP) Luminescent Assay

The CellTiter-Glo**^®^** 3D Cell luminescent assay (Promega, G7571, Anatech Analytical Technology, Bellville, South Africa) is a homogeneous method for the determination of cellular proliferation and quantification of ATP present in metabolically active cells. An equal volume (50 μL) of reconstituted ATP reagent and the cell suspension was mixed on a shaker for 2 min to induce cell lysis, followed by incubation at room temperature for 10 min in the dark to stabilize the luminescent signal. The luminescent signal was read using the 1420 multilabel counter victor3 (Perkin–Elmer, Separation Scientific, Johannesburg, South Africa).

### 2.7. Cytotoxicity-Lactate Dehydrogenase (LDH) Assay

The membrane integrity was assessed by estimating the amount of LDH present in the culture media. The cytosolic enzyme LDH will be released into the media due to membrane damage. The Cyto-Tox96 X assay (Anatech, Promega G 400) was used to measure the LDH released. An equal volume (50 μL) of reconstituted LDH reagent and cell culture medium was mixed and incubated in the dark at room temperature for 30 min. The colourimetric mixture was measured spectrophotometrically at 490 nm (Perkin–Elmer, VICTOR3™).

### 2.8. Caspase 3/7 Activity

A549 cells cultured in 3.4 cm diameter plates were incubated with cobalt complexes (**III** and **IV**) under standard culture conditions. After 24 h of treatment, cultured media from each group were transferred to white-walled luminescent plates. Cells from each plate were detached using TrypLE Express (Invitrogen, 12605-028, Johannesburg, South Africa), centrifuged and re-suspended in culture media. An equal volume of cell suspension was then transferred to their respective wells. 100 µL of Caspase-Glo**^®^** 3/7 Reagent was added to each well, mixed and incubated in dark for 1 h. The luminescence produced in each well was read using the 1420 multilabel counter victor3 (Perkin–Elmer, Separation Scientific).

### 2.9. Statistical Analysis

All the results were expressed as mean ± SEM. Statistical significance was determined using one way ANOVA followed by Dunnett’s multiple comparison tests. *p* < 0.05 was considered statistically significant.

## 3. Results

We have recently reported the urease-like activity of cobalt (III) complexes using urea as the ideal substrate [33]. In the present study, we have explored the preliminary anticancer activities of these urease mimetic cobalt (III) complexes towards lung cancer and breast cancer cell lines. Transition metal-based enzyme mimics are significant agents for several catalytic and therapeutic applications. They offer several advantages over the conventional enzymes, such as long shelf life, undemanding storage conditions and a wide window of activity, whilst maintaining the desired enzymatic activity. Thus, suitably designed transition metal complexes with biocompatible ligands as enzyme mimics can pave the way for a new era of therapeutic agents.

### 3.1. Synthesis, Characterization, Stability and Urease Mimetic Activity of the Complexes

**Figure 1:**
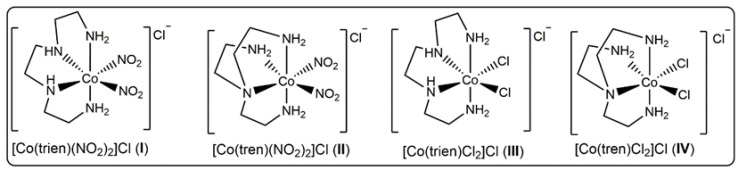
Structures of the complexes under study.

Urease activity is usually considered unfavourable for agricultural, industrial, and pharmaceutical applications [40,41,42]. Medicinally, urea is a common physiological metabolite and serves as an ideal substrate for pathogen growth. Therefore, urease inhibition has long been used as a convenient tool to target several pathogens in the human body [24,28,30,43]. However, lately, the notorious urease activity has been successfully employed in anticancer treatment. The alkalinity resulting from the urease catalysed hydrolysis of urea has been shown to be effective in treating cancer cells [14,19,44]. In the present study, we have reported the urease-like anticancer activity of urease mimetic cobalt (III) complexes (Figure 1) against lung cancer cells (A549), breast cancer cells (MCF7), and oesophageal cancer cells (HKESC-1). All the complexes were prepared in excellent yields following reported methods and characterized by various spectroscopic techniques [33,45,46].

The stability of the compounds in the cell culture medium was analysed by UV/Vis spectroscopy as well as by SC-XRD analyses. A solution of all the complexes was made (~7 mM, 1 mL) by dissolving the compounds in a solution of RPMI supplemented with 10% foetal bovine serum, 1% penicillin-streptomycin and 1% amphotericin-B. The resulting solutions were maintained at 37 °C, and their UV/Vis spectra were analysed for 3 days, every 12 h. There was no appreciable change in the spectra for the complexes over 3 days, indicating the stability of the complexes in solution (Figure S1). Furthermore, efforts were made to re-crystallise the compounds from a solution of the cell culture media by means of the slow evaporation method. Diffraction quality single crystals were obtained from the solutions of [Co(tren)(NO_2_)_2_]Cl (**II**) and [Co(trien)Cl_2_]Cl (**III**). The crystals were analysed by SC-XRD analyses and were found to be structurally similar to the initial compounds. The crystal structures are presented in Figures S2 and S3. Interestingly, while [Co(trien)Cl_2_]Cl was similar to the starting material, the [Co(tren)(NO_2_)_2_]Cl crystallised as a polymorph in a different space group. The most prevalent polymorph for this compound exists in orthorhombic space group (*Pbca*), and the only reported monoclinic form (*A*2) was isolated serendipitously [47]; however, the change from the orthorhombic to monoclinic could imply that the latter is the therapeutically active polymorph, and hence gets stabilised in the cell culture medium. Such preference for selective conformations is quite evident in biological systems [48,49]. Nevertheless, the crystals obtained after recrystallization were found to be structurally similar to the starting ones, further substantiating the stability of the compounds in the cell culture media.

The urease-like activity of some of the complexes was explored using urea as an ideal substrate, and the results were presented in recent publications [33,50]. The increase in the basicity of the solution is believed to result from catalytic cleavage of urea during the reaction. A detailed in vitro catalytic study on [Co(tren)Cl_2_]Cl (**IV**) induced cleavage of urea into ammonia was reported by some of us recently. The increasing alkalinity was monitored spectrophotometrically with the aid of a pH dependent indicator, phenolphthalein [33].

**Figure 2:**
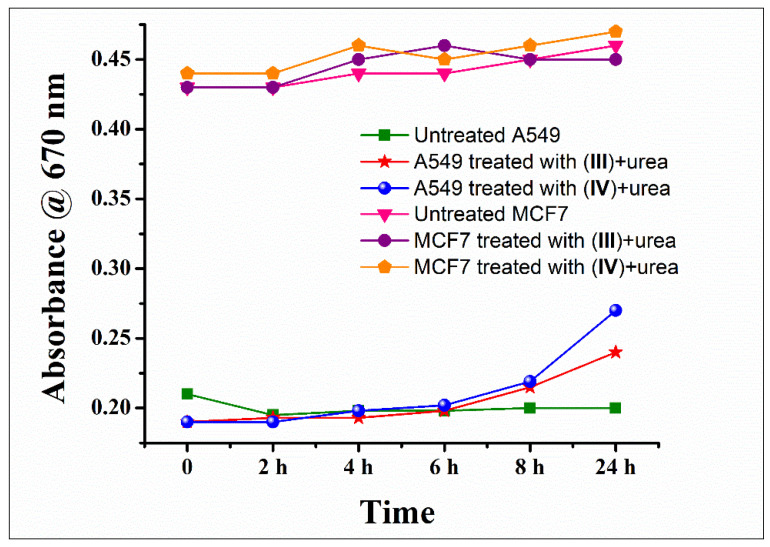
Urease mimetic activity of [Co(trien)Cl_2_]Cl (**III**) and [Co(tren)Cl_2_]Cl (**IV**) measured extracellularly in the cell culture medium. The absorbance at 670 nm corresponds to the ammonia concentration generated in response to the catalytic urease activity.

The ammonia generated because of urease mimetic activity in treatment groups was ascertained by pH measurements of the cell culture media after 24 h of treatment of A549 cells with the cobalt (III) complexes. Interestingly, the culture media from the cells treated with [Co(tren)Cl_2_]Cl (**IV**) and [Co(trien)Cl_2_]Cl (**III**) were found to be slightly basic as compared to the other groups. This prompted us to measure the urease mimetic activity of the chloro complexes under treatment conditions. Therefore, confluent cells in culture dishes were treated with the optimised dose of the cobalt (III) complexes (32 μM) in the presence of urea (2 mM). The results were compared to cells treated with urea alone (2 mM) (untreated group) to negate any background absorbance that may arise due to the culture medium or urea. A total of 100 μL of the culture medium was withdrawn at fixed time intervals, and the urease mimetic activity was measured using the urease activity kit from Sigma Aldrich as per the protocol available with the test kit. The increase in absorbance at 670 nm at fixed time intervals for the cobalt treated groups was compared to the untreated group. The results are presented in Figure 2. The A549 cells treated with [Co(tren)Cl_2_]Cl (**IV**) and [Co(trien)Cl_2_]Cl (**III**) depicted a gradual increase in absorbance at 670 nm, and after 24 h of treatment, the absorbance, and hence the urease mimetic activity for [Co(tren)Cl_2_]Cl (**IV**) was significantly higher than [Co(trien)Cl_2_]Cl (**III**). Similar trends have been reported for catalytic studies as well [33,50]. There was no appreciable change observed in the culture medium of untreated cells, thereby substantiating the urease mimetic generation of ammonia by the cobalt (III) complexes. The urease activity results are also reflected in the anticancer activities, with [Co(tren)Cl_2_]Cl (**IV**) performing better than [Co(trien)Cl_2_]Cl (**III**) for A549 cells.

The urease activity results of the cobalt (III) complexes for MCF7 cells were completely surprising and conflicting. There was no appreciable change in absorbance at 670 nm for the cells treated with [Co(tren)Cl_2_]Cl (**IV**) and [Co(trien)Cl_2_]Cl (**III**) over 24 h (Figure 2). The absorbances for the treated groups and the untreated group were almost the same, indicating no measurable urease activity in this case. Predictably, the complexes demonstrated poor anticancer activities as well towards MCF7 cells; however, the activity of the complexes against HKESC-1 cells was comparable to that observed in A549, negating the role of cell culture media in the observed therapeutic activity. Nevertheless, it is to be noted that urease induced alkalization and anticancer activity has been reported in both A549 and MCF7 cells [12,18].

### 3.2. Cytotoxicity-Lactate Dehydrogenase (LDH) Assay

**Figure 3:**
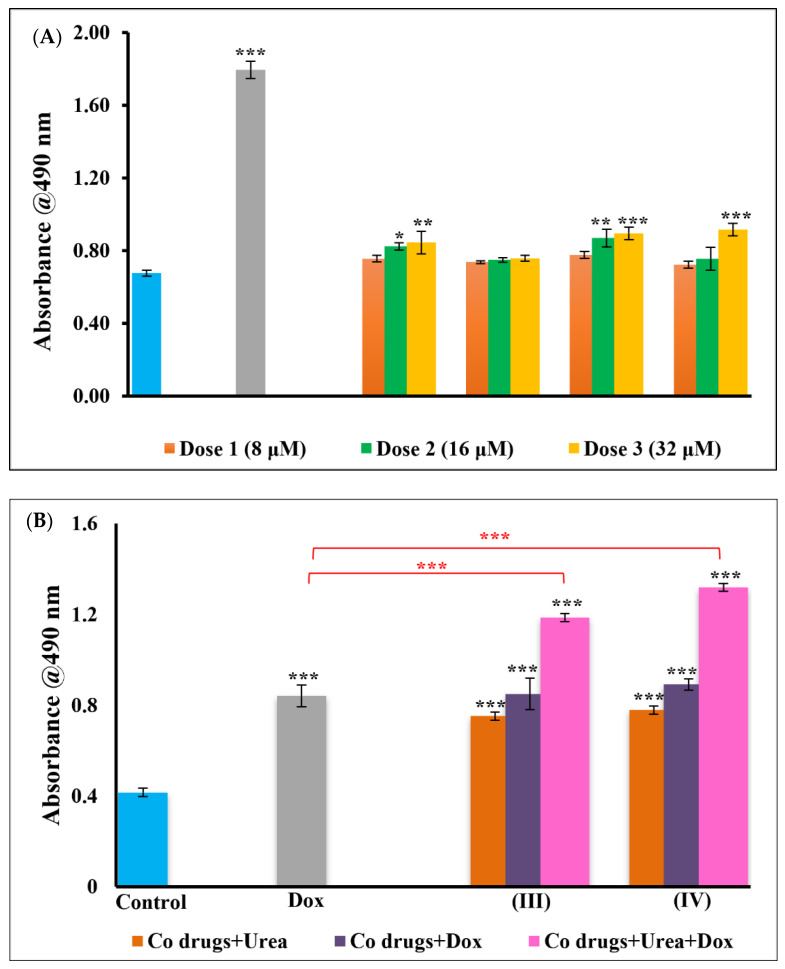
LDH levels of A549 cells. (**A**) The control cells (untreated) and cells treated with different concentrations (8 μM, 16 μM, and 32 μM) of the cobalt (III) complexes and doxorubicin (50 nM) showed different levels of LDH release. (**B**) Significant LDH release was observed when the optimized dose of the cobalt complexes (32 μM) was combined with urea (2 mM) and doxorubicin (50 nM) in treated groups. The action of doxorubicin was fairly increased when the complexes (**III** and **IV**) were combined with urea. Loss of membrane integrity and LDH release can be directly related to pH increase and facilitating Doxorubicin into cells. The data represent the mean ± SEM. Results are significantly different at * *p* < 0.05, ** *p* < 0.01 and *** *p* < 0.001 when compared to control and doxorubicin.

The WS1 cells in the control and treated groups were seen to be healthy with no significant difference observed in growth and proliferation (Figure S4). The LDH content quantified from these groups did not show a significant difference indicating the safety of the standardized doses. In contrast, the membrane integrity of A549 cells after the treatment with the cobalt complexes depicted significant damage and release of LDH into the culture media. It was interesting to note that the degree of membrane damage in cells treated with different Cobalt complexes varied in accordance with the urease mimetic activity of the complexes. Here, [Co(trien)Cl_2_]Cl (**III**) and [Co(tren)Cl_2_]Cl (**IV**) complexes outperformed other Co complexes (Figure 3A). The [Co(tren)(NO_2_)_2_]Cl (**II**) and [Co(trien)(NO_2_)_2_]Cl (**I**) complexes exhibit poor catalytic urease activity as well, owing to the strong affinity and poor lability of the nitro group towards cobalt [33].

Based on these preliminary results, further experiments were performed using the two chloro substituted complexes, [Co(trien)Cl_2_]Cl (**III**) and [Co(tren)Cl_2_]Cl (**IV**), while the nitro complexes were dropped out. To ascertain the urease-like anticancer activity of the cobalt complexes, the cells were treated with the complexes alone and with the addition of urea. The resulting alkalinity of urease activity has been shown to enhance the activity of weakly basic anticancer drugs, such as doxorubicin. Such drugs–enzyme combinations and conjugates are gaining increasing attention in recent times [19,36]. Therefore, we also studied the combined effects of the cobalt complexes and doxorubicin against A549 cells. The IC_50_ dose for doxorubicin was used for all the experiments. There was a significant dose dependent LDH release with increasing concentrations of the cobalt complexes and their standardized dose combinations. Interestingly, the activity of the cobalt complexes improved on the addition of urea compared to the complexes alone. When the complex–urea combination was used with doxorubicin, the activity increased by several folds compared to the cobalt complexes alone or with urea (Figure 3B). Moreover, the combination groups also surpassed the toxicity induced by the positive control drug, doxorubicin (Figure 3B) alone. This substantiates our hypothesis that the cobalt complexes mediate the hydrolysis of urea in the culture medium, thereby increasing the pH and altering the tumour microenvironment. The increased basicity also improves the diffusion and the activity of doxorubicin against cancer cells. The results show the following trend; complex alone < complex + urea < doxorubicin < complex + doxorubicin < complex + urea + doxorubicin. There was no appreciable effect when MCF7 were treated with the dose optimised for A549 (Figure S5). However, complexes (**III** and **IV**) were found to be toxic to HKESC-1 cells (Figure S6).

### 3.3. Adenosine Triphosphate (ATP) Cell Metabolism Assay

**Figure 4:**
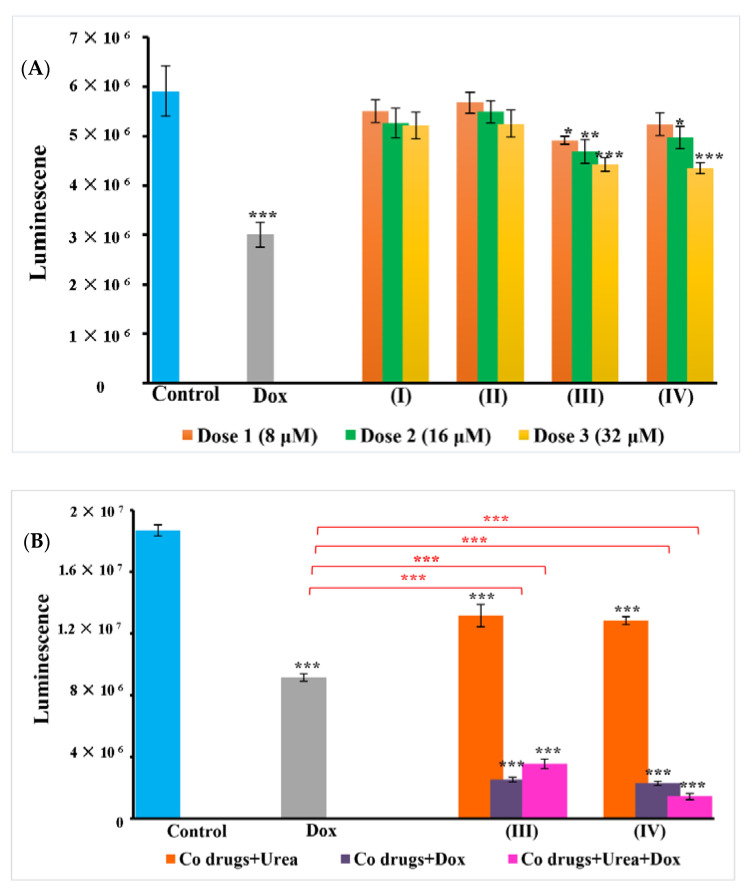
ATP metabolism in A549 cells. (**A**) The cellular metabolism was accessed with the ATP levels in the control (untreated) and cells treated with different concentrations (8 μM, 16 μM, and 32 μM) of the cobalt complexes. (**B**) Standardized doses of complex (32 μM) combination with urea (2 mM) and doxorubicin (50 nM). The cytotoxic ability is evident in the figure with decreased levels of ATP in cobalt (III) complexes treated groups. As seen in the figure, complexes combination with urea and doxorubicin profound decrease in ATP. (**A**,**B**) clearly depict the difference in the action of cobalt complexes alone and in combination with doxorubicin and urea. Here, the cytotoxic activity and decrease in ATP levels of complexes (**III** and **IV**) treated groups signify their ability to boost the action of doxorubicin against A549 cells. The data represent the mean ± SEM. Results are significantly different at * *p* < 0.05, ** *p* < 0.01 and *** *p* < 0.001 when compared to control and doxorubicin.

A similar trend was observed with ATP of WS1 and A549 cells. No significant ATP proliferation was observed in WS1 cells depicting the safety of complexes against normal cells (Figure S7); however, toxicity induced by cobalt complexes in A549 was clearly observed in treatment groups. The dose dependent ability of the cobalt complex and their combinations in disrupting cellular membrane and release of LDH was equally observed in the ATP metabolism of cells. There was an increase in cellular metabolism, as demonstrated by the increased levels of ATP in A549 control cells. Exposure of A549 cells to the cobalt (III) complexes reduced the intracellular ATP pool. The rate of cellular proliferation and ATP levels were also altered after the treatment compared to control cells. The drugs ([Co(trien)Cl_2_]Cl (**III**) and [Co(tren)Cl_2_]Cl (**IV**))–urea combination as well as the combinations with doxorubicin, induced profuse toxicity in cells and reduction of ATP. These findings further support our claim that urease mimetic activity of the cobalt complexes induces anticancer effects by facilitating doxorubicin action, as observed in the LDH assay. The results are presented in Figure 4. As observed with LDH results, the complexes were found to be unresponsive towards MCF7 cells as compared to the untreated control, as well as doxorubicin. (Figure S8). In contrast, a similar activity trend was observed in HKESC-1 with increased ATP in complex + urea + doxorubicin groups (Figure S9).

### 3.4. Cellular Morphology

**Figure 5:**
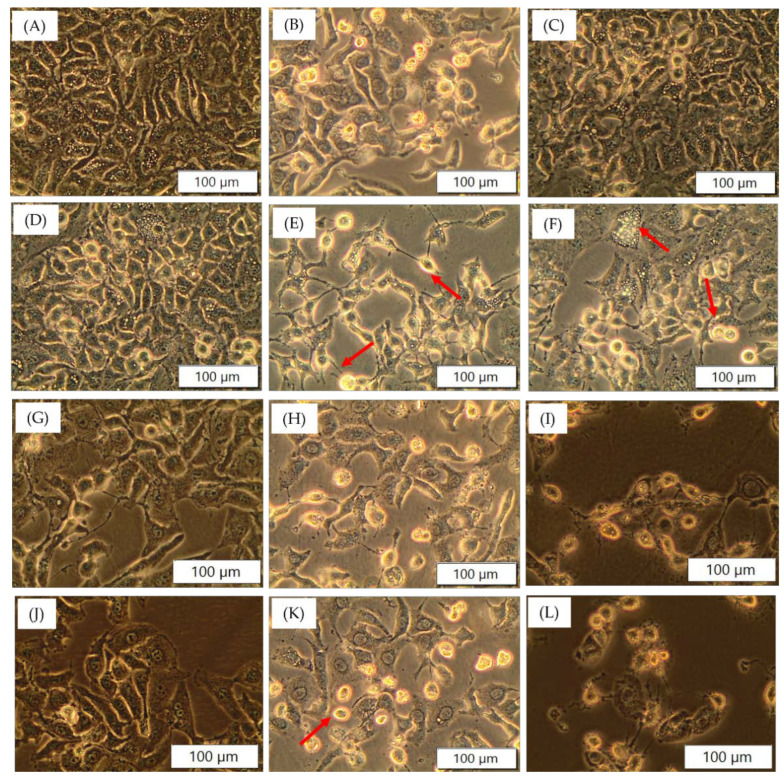
Morphology of A549 treated with cobalt complexes, urea and doxorubicin. (**A**) Control (untreated); (**B**) Dox (50 nM); (**C**) 32 μM (**I**); (**D**) 32 μM (**II**) (**E**) 32 μM (**III**); (**F**) 32 μM (**IV**); (**G**) 32 μM (**III**) +2 mM urea; (**H**) 32 μM (**III**) + 50 nM Dox; (**I**) 32 μM (**III**) + 2 mM urea + 50 nM Dox; (**J**) 32 μM (**IV**) +2 mM urea; (**K**) 32 μM (**IV**) + 50 nM Dox; (**L**) 32 μM (**IV**) + 2 mM urea + 50 nM Dox. Intact cellular morphology is seen in the control group compared to treated groups. Clear and evident round cells detached from culture plate (indicated with arrows) can be seen in the image. Vacuole formation (arrow in (**D**)), shrinkage of cells, fewer cell population indicates low cell proliferation and increased cell death. These signs of cell death are more prominent in group (**I**,**L**) where the complexes in presence of urea might have facilitated cytotoxic action of doxorubicin.

The A549 cells which were incubated with the cobalt complexes alone and in combination with urea and doxorubicin for 24 h showed distinct morphology in a concentration dependent manner compared to the control. The cells showed signs of cell death, with deformed membrane integrity, losing inter-cellular contact, vacuolation, rounding of cells and detachment from the culture plates (Figure 5). The toxicity of complexes on HKESC-1 cells was evident in morphology images (Figure S11); however, unlike A549, MCF7 cells did not respond well to the treatment (Figure S10).

### 3.5. Trypan Blue Viability Assay

**Figure 6:**
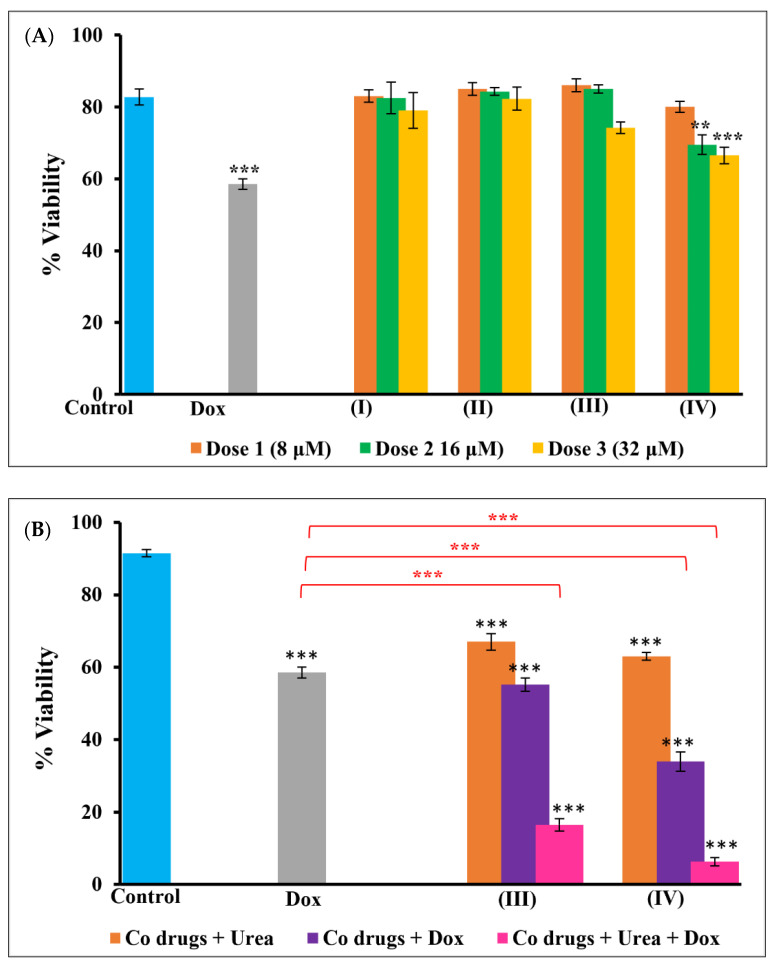
Trypan blue viability test in A549 cells. Graph (**A**) shows the viability of A549 cells treated with complex alone and in combination with doxorubicin. Graph (**B**) shows the commendable increase in the ability of doxorubicin in inducing cell death when combined with urea. As seen in other assays, cobalt **III** and **IV** complexes induced better toxicity in A549 cells compared to other complexes as seen in (**A**). Moreover, urea and doxorubicin induced more cell death whereby these cells stained well with trypan blue. Significance in these groups can be seen increased by several folds. Results are significantly different at ** *p* < 0.01 and *** *p* < 0.001 when compared to control and doxorubicin.

As observed in LDH and ATP assays, cell viability significantly reduced in A549 cells on treatment with the cobalt complexes and the urea and doxorubicin combinations (Figure 6). The cells were quantified as dead with their ability to take up trypan blue dye due to membrane disruption and permeability. The maximum number of dead cells was observed in complex combination with urea and doxorubicin compared to other treatment groups. It was interesting to note that all the assay results complemented each other, signifying the importance of drug combination and the role of cobalt (III) complexes in modifying cancer cell microenvironment in enhancing cell death.

### 3.6. Caspase 3/7 Activity

**Figure 7:**
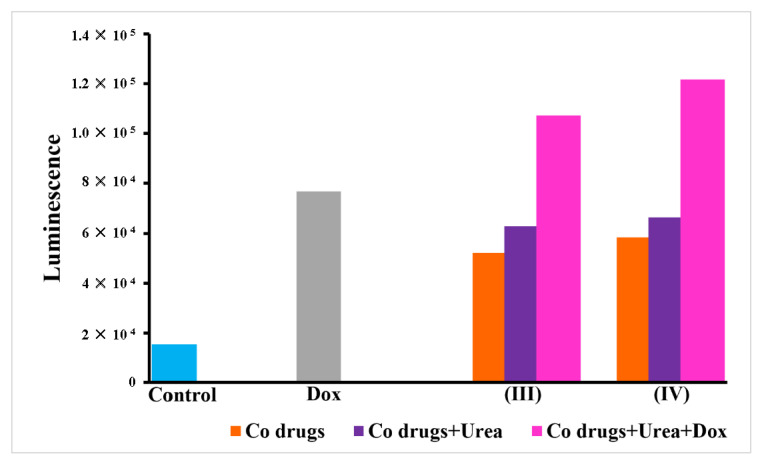
Caspase 3/7 activity in treated A549 cells. The figure shows comparative caspases activity in A549 cells treated with doxorubicin alone and in combination with cobalt complexes and urea. An increase in luminescence and thus enhanced caspase 3/7 activity is clear in the combination group.

Caspase 3/7 is one of the important executioner cascades leading to apoptosis. The results can be promptly seen in cobalt complexes (**III** and **IV**) in combination with urea and doxorubicin. The luminescent signals elevated in these groups compared to control and authenticate the role of caspases in inducing cell death (Figure 7).

## 4. Discussion

Currently, cancer research is dominated by the need and design of target specific anticancer drugs with minimal side effects. One way to achieve this is to alter the tumour microenvironment. The acidic tumour microenvironment plays a pivotal role in the growth and sustenance of tumour cells by inducing resistance and T-cell stasis, thereby assisting the tumour to evade the self-immune mechanism [20,51]. The acidity in the microenvironment also results in the localization of basic drugs in the extracellular matrix, thereby reducing the effective drug dosage. This often results in poor activity and drug resistance [20]. In the present study, we have explored an approach for indirectly altering the tumour microenvironment by urease mimetic cobalt (III) complexes. We have presented the preliminary results of the cytotoxicity of urease mimetic cobalt (III) complexes alone, and in combination with a weakly basic drug, doxorubicin. The complexes, on their own exhibit weak cytotoxicity towards A549 cells. This activity is enhanced by many folds by introducing 2 mM urea to the cell culture medium. The concentration of urea used in the present study was decided based on literature reports. The cytotoxicity of urea towards A549 cells was reported by Wong et al. to be 13 mM in the presence on 2 U/mL of urease. Urea alone was shown to be non–toxic up to 40 mM concentrations [19]. Therefore, the present work was performed by introducing a very low concentration of urea in the cell culture medium which itself has shown a significant role in promoting cell death of A549 cells in this study. The cobalt (III) complexes hydrolyse urea generating ammonia and bicarbonate, and consequently raising the pH in the surrounding microenvironment. The urease mimetic activity of the cobalt complexes was measured extracellularly in the cell culture medium. The efficacy of the cobalt drugs was also tested against MCF7; however, unpredictably, the observed cytotoxicity was poor against MCF7, as compared to that observed against A549. The selective activation of the complexes in the presence of A549, but not MCF7 was quite intriguing. Initially, we postulated that the culture media used for the growth and maintenance of the cells play a role in the activity of the complexes since all other parameters were constant during the studies. A549 cells were grown in RPMI base media and MCF7 cells were grown in DMEM. Possibly, the cobalt complexes interact with the constituents of the culture media and are rendered inactive or inert in DMEM, which results in loss of catalytic and anticancer activity in vitro. To further understand the role of cell culture media in the observed anticancer activity, and to rule out the cell line specificity of the proposed cobalt drugs, the cytotoxicity of the complexes was also investigated against HKESC-1 cells grown in complete DMEM media. As reported in Figures S6, S9 and S11, the cobalt drugs displayed excellent cytotoxic activity against the oesophageal cancer cells. Moreover, the proposed combination treatment of the cobalt complexes with doxorubicin surpassed the activity of doxorubicin alone, as also seen in A549. Hence, at this stage, we can refute the role of cell culture media in activation and/or deactivation of the cytotoxic activity of the cobalt complexes; however, the exact mechanism of inactivation towards MCF7 cannot be predicted unambiguously at this stage. The complexes might have a higher dose against MCF7. Nevertheless, these results further substantiate our hypotheses, that the complexes exert their anti–cancer action via urease mimetic activity; however, future studies are warranted in vivo and in clinical models to establish the role of these complexes on different types of cancers.

The trends observed for the urease mimetic activity were also reflected in the anticancer activities. The generated ammonia as well as raised pH upsurges the cytotoxicity of the cobalt (III) complexes towards cancer cells. Such altering of the microenvironment can also be manipulated to enhance the activity of weakly basic drugs. Under acidic conditions, basic drugs, such as doxorubicin, tend to sequester in the acidic extracellular environment, thus failing to diffuse into the hydrophobic cell membrane. This often results in loss of therapeutic activity and drug resistance. In this study, we report a combination therapy of the cobalt (III) complexes with doxorubicin. The combination is proposed to be working synergistically, wherein the cobalt (III) complexes hydrolyse urea to generate ammonia, thus neutralizing the acidity in the tumour microenvironment. This subsequently, enhances the diffusion of doxorubicin into the cells, thereby, improving the overall cytotoxic activity. The cobalt complexes do not directly target the cancer cells; rather, they act on the extracellular microenvironment, disrupting the physiological factors that enable tumour growth. Thus, they exert a target specific cytotoxic action controlled by the surrounding pH. This can also be attributed to the low toxicity of the complexes towards normal cells that operate under strictly controlled pH conditions. To further confirm the mechanism of apoptotic action, caspase 3/7 expression was determined in treated groups. Even though the cell death stimulus induced by caspase 3 and 7 differs, they are yet considered universal executioner caspases [52,53,54]. Activation of these caspases leads to cell shrinkage, chromatin condensation, formation of apoptotic bodies of which a few significant signs can be seen in morphology images of A549. Unlike normal cells, metabolic pathways in cancer tissues are different and play a crucial role in maintaining their uncontrolled growth and proliferation. Mitochondria, being the key regulator of metabolic activities and cell cycle, has emerged as one of the important targets for targeting cancer tissues for improved therapy [55]. Studies focusing on oxygen consumption rate (OCR) and extracellular acidification rate (ECAR) are also known to contribute to cellular pH through mitochondrial glycolysis and respiration [56]. Similarly, Reactive Oxygen Species (ROS) have been well studied in regulating cell death in cancer. Mitochondria, as the major site of Iron-dependent peroxidation of lipids, generates excessive ROS leading to ferroptosis, a necrotic cell death pathway [57]. Hence, the role of mitochondria in targeted cancer therapy is emerging as a promising research area that may contribute to significant outcomes in studies with similar interests. The tumour microenvironment has shown to play pivotal roles in maintenance of tumour heterogeneity and subsequent cancer metastasis. Therefore, more research is now being directed against the tumour microenvironment along with the cancer cells. There are many proposed methods of targeting the tumour microenvironment, one of which involves neutralization of the acidosis in the microenvironment to improve the diffusion of chemotherapeutic drugs. Currently, a few combination therapies are being tried clinically, namely, a combination of acetazolamide and radiation therapy for small cell lung cancer treatment. This approach targets the carbonic anhydrase enzyme along with the cancer cells [58]. Other reports involve the combination of the isolated and purified urease enzyme extract with drugs such as doxorubicin [12,22,39,44,51]. Herein, we report the first combination of using a basic anticancer drug with synthetic urease mimetic transition metal complexes. The results suggest admirable synergism, which can combat drug resistance as well. Nevertheless, these are preliminary results that warrant further investigation into developing effective and nontoxic anticancer therapy.

## 5. Conclusions

Urease activity was long viewed as detrimental, and research was mainly focused on inhibiting the enzyme’s activity; however, the undesirable urease activity can be utilized to neutralize the acidity in tumour microenvironments, thereby leading to enhanced therapeutic efficiency. We have presented the preliminary results of the cytotoxicity induced by urease mimetic cobalt (III) complexes on lung cancer cells. The cytotoxicity results correlate well with the observed urease mimetic activity. The complexes reported in this study are found to be nontoxic towards normal cells, making them an ideal drug candidate for treating cancer cells. To further understand the application of these complexes as anticancer drugs, their dose responses on MCF-7 and HKESC-1 were also studied. With moderate toxicity on MCF-7 but commendable action against HKESC-1 cells, it can be concluded that the active complexes (III and IV) were not cell line specific and further research is warranted to understand the reason behind these specificities. Moreover, synthetic enzyme mimics based on transition metal complexes serve several advantages over the actual enzyme; they are robust, with long shelf life, and often can greatly reduce the cost of manufacturing. The urease, like the action of the cobalt (III) complexes, also improves the therapeutic activity of weakly basic drugs, such as doxorubicin, by inducing apoptosis as seen by the expression of caspase3/7 in treated groups; however, the conflicting results obtained with MCF7 cell lines warrant further investigation using in vivo and clinical models. The concept introduced in this article is worthwhile and deserves further investigation.

## Data Availability

Not applicable.

## Supplementary Materials

The following supporting information can be downloaded at: https://www.mdpi.com/article/10.3390/pharmaceutics14010211/s1, Characterization data for all the cobalt complexes. Figure S1: UV/Vis spectra of the cobalt (III) complexes in the cell culture media (RPMI supplemented with 10% foetal bovine serum, 1% Penicillin-streptomycin, and 1% amphotericin-B) displaying the stability of complexes in the cell culture media, Figure S2: Molecular structure of [Co^III^(trien)Cl_2_]Cl·H_2_O with displacement ellipsoids drawn at 50% probability, showing the atomic numbering scheme where the heteroatoms have been labelled only for clarity. Dashed red and green lines indicate hydrogen bonds. The crystals were grown by slow evaporation of a solution of [Co(trien)Cl_2_]Cl in RPMI supplemented with 10% foetal bovine serum, 1% Penicillin-streptomycin, and 1% amphotericin-B, Figure S3: Molecular structure of [Co^III^(tren)(NO_2_)_2_]Cl with displacement ellipsoids drawn at 50% probability, showing the atomic numbering scheme where the heteroatoms have been labelled only for clarity. Dashed red and green lines indicate hydrogen bonds. The crystals were grown by slow evaporation of a solution of [Co(tren)(NO_2_)_2_]Cl in RPMI supplemented with 10% foetal bovine serum, 1% Penicillin-streptomycin, and 1% amphotericin-B, Figure S4: LDH levels of WS1 cells treated with 32 μM of each of the cobalt complexes. The results were non-significant at *p* < 0.05 compared to control representing the safety of the Co (III) complexes against WS1 cells, Figure S5: LDH levels of MCF7 cells. [Co drugs]: 32 μM, [urea]: 2mM, [Dox]: 50 nM, Figure S6: LDH levels of HKESC-1 cells. [Co drugs]: 32 μM, [urea]: 2mM, [Dox]: 50 nM, Figure S7: ATP metabolism in WS1 cells treated with 32 μM of each of the cobalt complexes. The results were non-significant at *p* < 0.05 compared to control representing the safety of the Co (III) complexes against WS1 cells, Figure S8: ATP proliferation of MCF7 cells. [Co drugs]: 32 μM, [urea]: 2mM, [Dox]: 50 nM, Figure S9: ATP proliferation of HKESC-1 cells. [Co drugs]: 32 μM, [urea]: 2mM, [Dox]: 50 nM, Figure S10: Morphology of MCF7 treated with cobalt complexes, urea and doxorubicin. (**A**) Control; (**B**) Dox (50 nM); (**C**) 32 μM (III); (**D**) 32 μM (IV); (**E**) 32 μM (III) +2 mM urea; (**F**) 32 μM (**IV**) + 2 mM urea; (**G**) 32 μM (**III**) +2 mM urea + 50 nM Dox; (H) 32 μM (**IV**) +2 mM urea + 50 nM Dox, Figure S11. Morphology of HKESC-1 treated with cobalt complexes, urea and doxorubicin. (**A**) Control; (**B**) Dox (50 nM); (**C**) 32 μM (**III**); (**D**) 32 μM (**IV**); (**E**) 32 μM (**III**) + 2mM urea; (**F**) 32 μM (**IV**) + 2 mM urea; (**G**) 32 μM (**III**) + 2 mM urea + 50 nM Dox; (H) 32 μM (**IV**) + 2 mM urea + 50 nM Dox.
