# Supplementary material for: Anticancer Activity of Urease Mimetic Cobalt (III) Complexes on A549-Lung Cancer Cells: Targeting the Acidic Microenvironment
Source: Pharmaceutics. 2022 Jan 17;14(1):211. doi: 10.3390/pharmaceutics14010211 (PMC8780642; doi:10.3390/pharmaceutics14010211)
Supplement: Supplementary file 1 [file pharmaceutics-14-00211-s001.zip › pharmaceutics-1484555-supplementary.pdf]

pharmaceutics

MDPI

Supplementary Materials: Anticancer Activity of Urease Mimetic Cobalt (III) Complexes on A549-Lung Cancer Cells: Targeting the Acidic Microenvironment

Bhawna Uprety, Rahul Chandran, Charmaine Arderne and Heidi Abrahamse

Characterization of the complexes

[Co(trien)(NO_2)$_2$]Cl (**I**)*:* Yield: 69.1%. Selected IR bands (vmax, cm^{-1}): v(N-H): 3274, 3094; v(N-O) 1615, 1583; v(CH2) 1474, 1406; v(NO2) 1342, 1304; v(C-N) 1156; v(NH) 820, 827; v(CH2) 747. ^{1}H NMR (D_2O, 400MHz): δ (ppm) 2.96 (br s, 6H, -CH_2-CH_2-), 3.57 (br s, 1H, NH-CH_2-CH_2-NH_2). 4.79 (br s, 5H, NH-CH_2-CH_2-NH_2). ^{13}C NMR (D_2O + CH_3OD, 400 MHz): δ (ppm) 56.84, 54.81, 41.99.
[Co(tren)(NO_2)$_2$]Cl (**II**)*:* Yield: 67.3%. Selected IR bands (vmax, cm–1): v(N–H): 3281, 3156, 3091; v(N–O) 1589; v(CH_2) 1474; v(NO_2) 1339, 1304; v(C–N) 1146; v(NH) 827; v(CH_2) 745. UV-Vis (H_2O): 438 nm (ε, 46.33 M^{-1} cm^{-1}), 322 nm (ε, 788 M^{-1} cm^{-1}). ^{1}H NMR (D_2O, 400MHz): δ (ppm) 3.48–3.24 (m, 8H, –N–CH_2–CH_2–N–), 3.08–3.01 (m, 4H, –N–CH_2–CH_2–NH_2). ^{13}C NMR (D_2O + CH_3OD, 400 MHz): δ (ppm) 62.46, 60.22, 44.43.
[Co(trien)Cl)$_2$]Cl (**III**)*:* Yield: 94.9%. Selected IR bands (vmax, cm^{-1}): v(N-H): 3261, 3194, 3091; v(CH2) 1617, 1568; v(C-N) 1165, 1108, 1054; v(NH) 879; v(CH2) 795. ^{1}H NMR (D_2O, 400MHz): δ (ppm) 2.51–2.78 (m, 4H, -NH-CH_2-CH_2-NH-), 2.93–3.52 (m, 8H, NH_2-CH_2-CH_2-NH), 5.61–5.91 (m, NH_2, NH). ^{13}C NMR (D_2O + CH_3OD, 400 MHz): δ (ppm): 57.28, 54.06, 43.05.
[Co(tren)Cl_2]Cl (**IV**)*:* Yield: 90%. Selected IR bands (vmax, cm^{-1}): v(N–H): 3234, 3135, 3081; v(CH_2) 1591, 1480; v(C–N) 1160, 1032; v(NH) 896; v(CH_2) 743. UV-Vis (H_2O): 533 nm (ε, 561 M^{-1} cm^{-1}), 370 nm (ε, 385 M^{-1} cm^{-1}). ^{1}H NMR (D_2O, 400MHz): δ (ppm). 3.60–3.56 (m, 2H, –N–CH_2–CH_2–N–), 3.39–3.32 (m, 4H, –N–CH_2–CH_2–N–), 3.25–3.22 (t, 2H, –N–CH_2–CH_2–N–), 2.86–2.82 (m, 2H, –N–CH_2–CH_2–N–), 2.74–2.70 (t, 2H, –N–CH_2–CH_2–N–), 5.46 (br s, NH_2, NH). ^{13}C NMR (D_2O + CH_3OD, 400 MHz): δ (ppm) 61.04, 60.00, 44.63, 43.47.

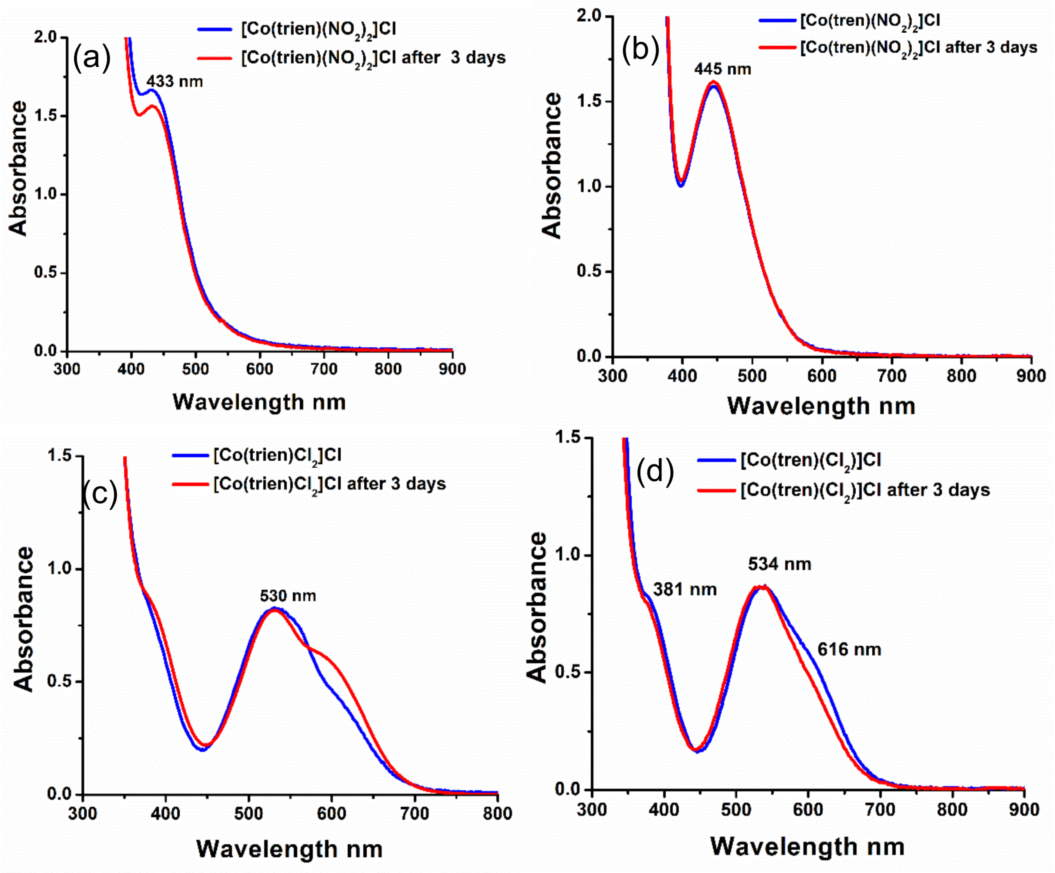

Figure S1. UV/Vis spectra of the cobalt (III) complexes in the cell culture media (RPMI supplemented with 10% foetal bovine serum, 1 % Penicillin-streptomycin, and 1% amphotericin-B) displaying the stability of complexes in the cell culture media.

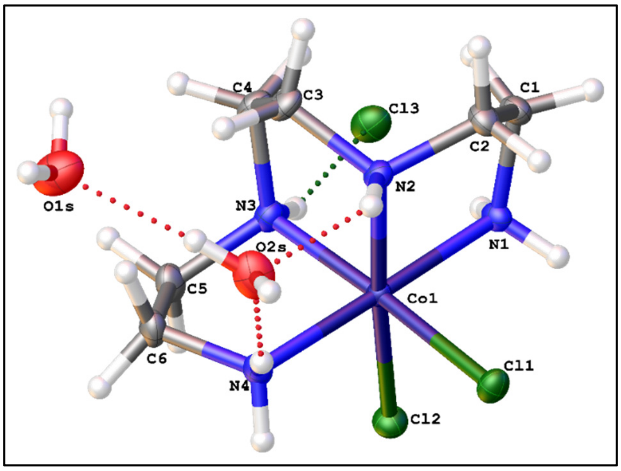

Figure S2. Molecular structure of $[Co^{III}(trien)Cl_2]Cl{\cdot}H_2O$ with displacement ellipsoids drawn at 50% probability, showing the atomic numbering scheme where the heteroatoms have been labelled only for clarity. Dashed red and green lines indicate hydrogen bonds. The crystals were grown by slow evaporation of a solution of $[Co(trien)Cl_2]Cl$ in RPMI supplemented with 10% foetal bovine serum, 1% Penicillin-streptomycin, and 1 % amphotericin-B.

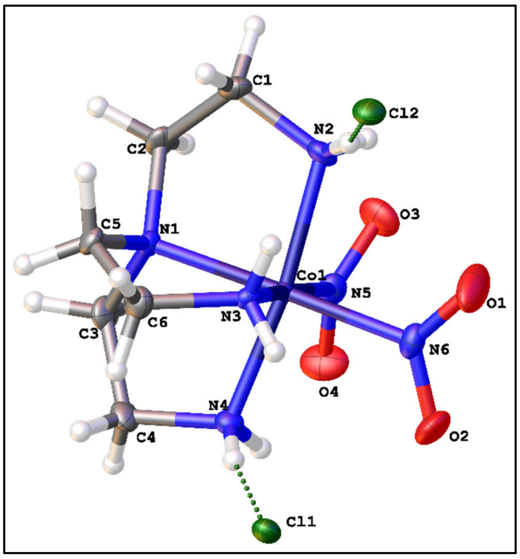

Figure S3. Molecular structure of $[Co^{III}(tren)(NO_2)_2]Cl$ with displacement ellipsoids drawn at 50% probability, showing the atomic numbering scheme where the heteroatoms have been labelled only for clarity. Dashed red and green lines indicate hydrogen bonds. The crystals were grown by slow evaporation of a solution of $[Co(tren)(NO_2)_2]Cl$ in RPMI supplemented with 10% foetal bovine serum, 1% Penicillin-streptomycin, and 1% amphotericin-B.

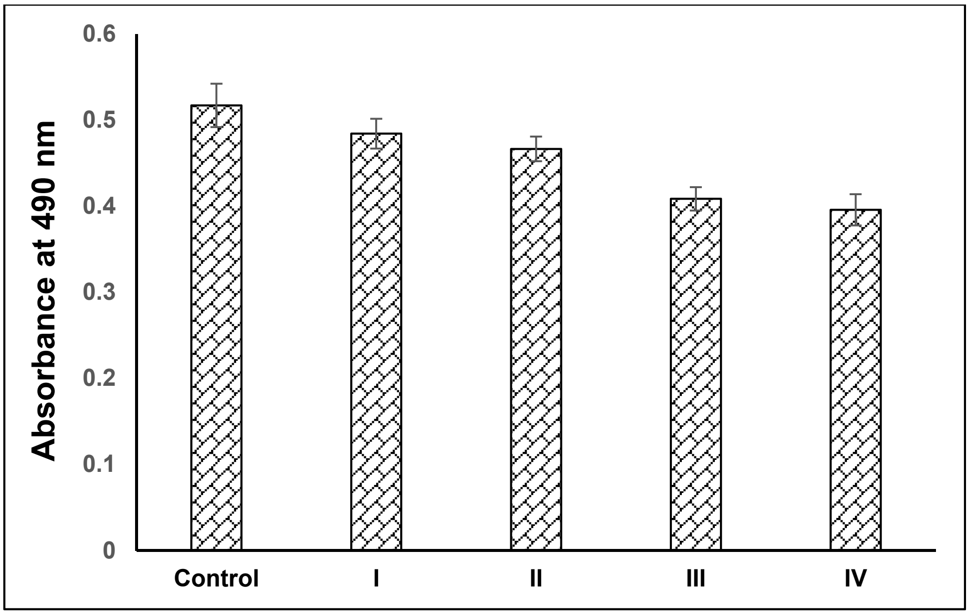

Figure S4. LDH levels of WS1 cells treated with 32 μM of each of the cobalt complexes. The results were non-significant at $p < 0.05$ compared to control representing the safety of the Co (III) complexes against WS1 cells.

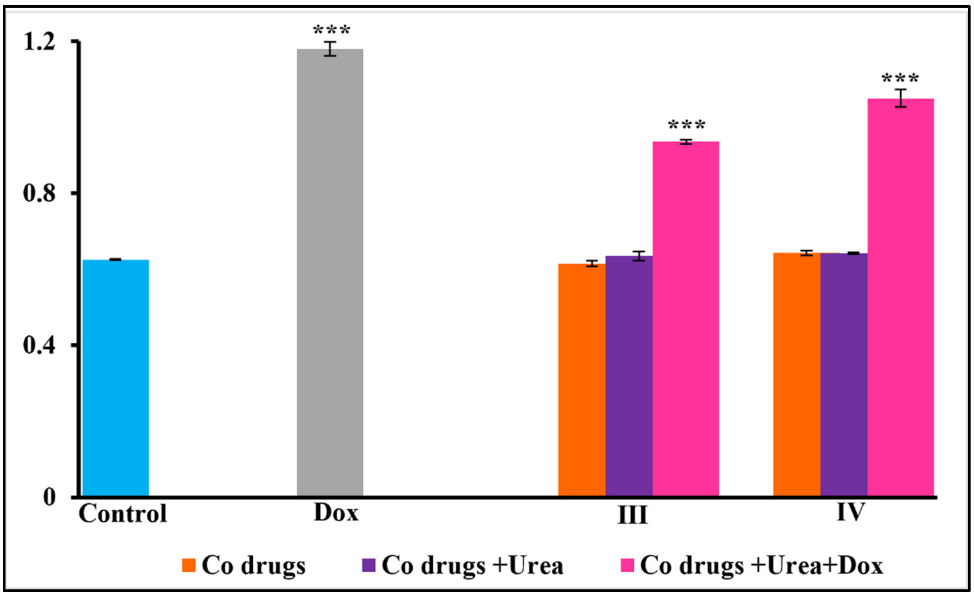

Figure S5. LDH levels of MCF7 cells. [Co drugs]: 32 μM, [urea]: 2mM, [Dox]: 50 nM. Increase in LDH levels can be seen in combination groups compared to control but not as effective as Doxorubicin. The data represent the mean ± SEM. Results are significantly different at *** $p < 0.001$ when compared to control and doxorubicin.

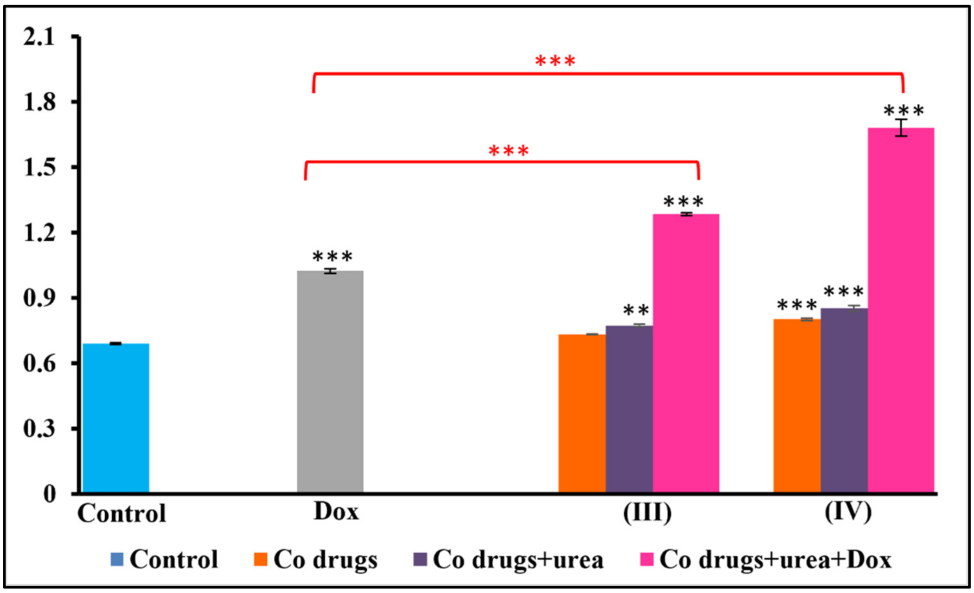

Figure S6. LDH levels of HKESC-1 cells. [Co drugs]: 32 µM, [urea]: 2mM, [Dox]: 50 nM. Prominent increase in LDH levels can be clearly seen here in groups treated with complex combinations compared to control and doxorubicin. The data represent the mean ± SEM. Results are significantly different at ** $p < 0.01$ and *** $p < 0.001$ when compared to control and doxorubicin.

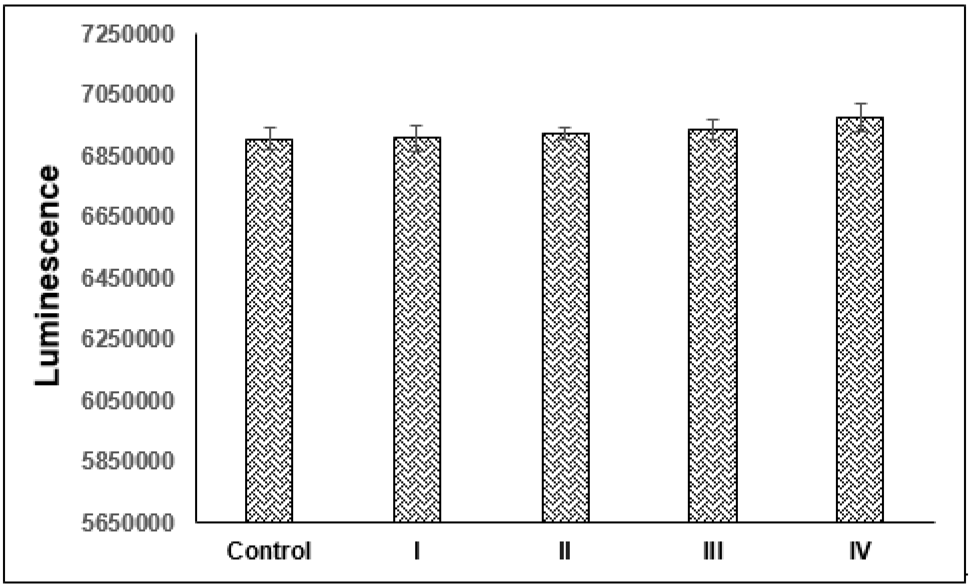

Figure S7. ATP metabolism in WS1 cells treated with 32 µM of each of the cobalt complexes. The results were non-significant at $p < 0.05$ compared to control representing the safety of the Co (III) complexes against WS1 cells.

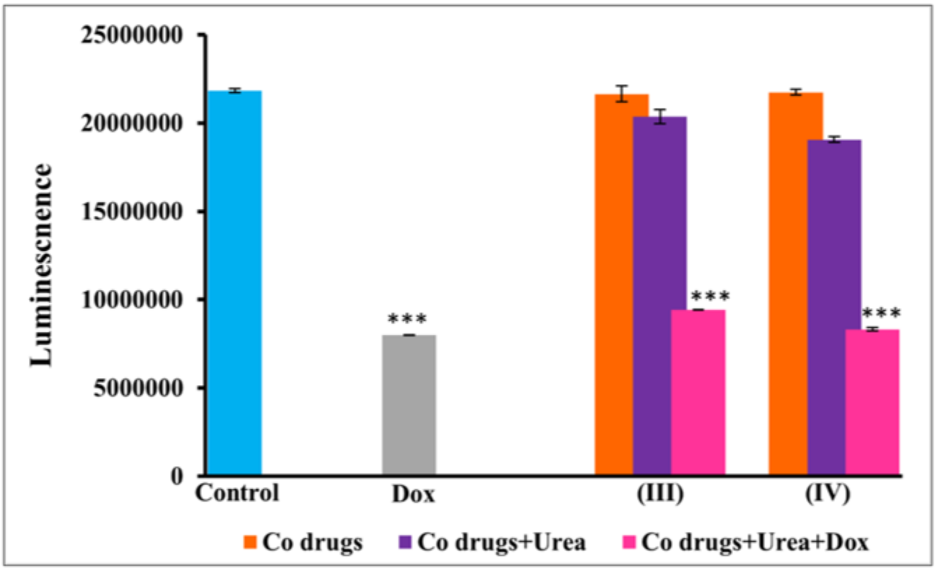

Figure S8. ATP proliferation of MCF7 cells. [Co drugs]: 32 μM, [urea]: 2mM, [Dox]: 50 nM. Similar effects can be seen as observed in LDH assay with more cell death induced by complex combinations thereby lowering ATP levels.

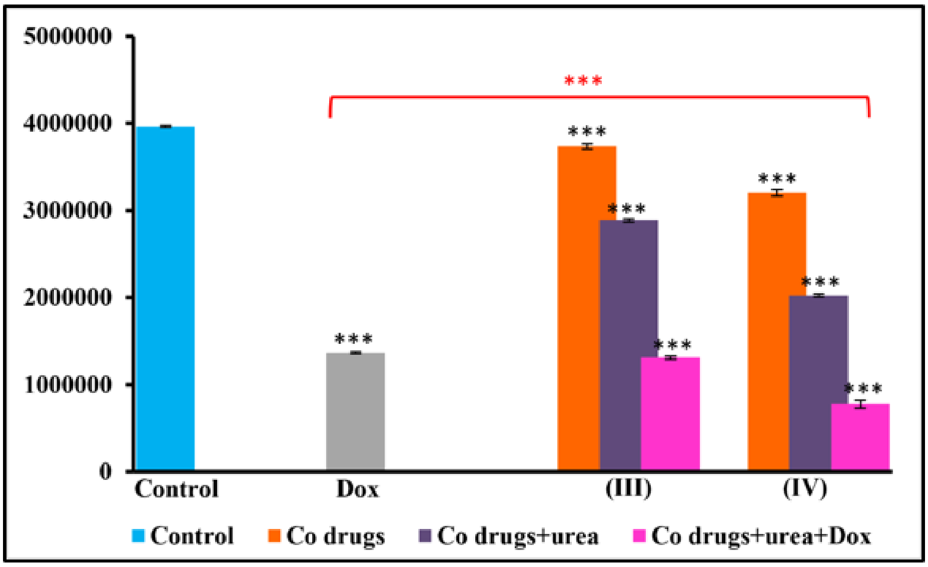

Figure S9. ATP proliferation of HKESC-1 cells. [Co drugs]: 32 μM, [urea]: 2mM, [Dox]: 50 nM.

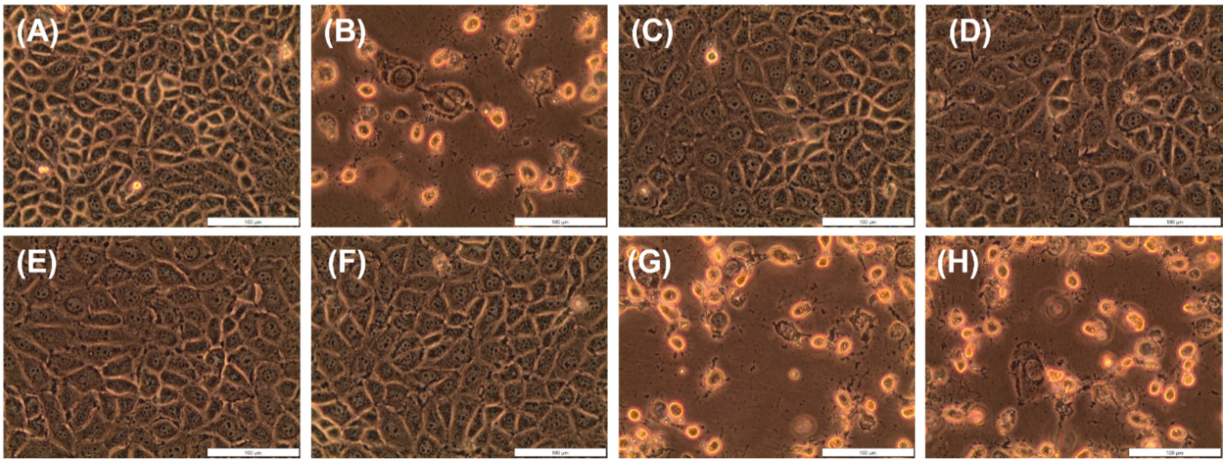

Figure S10. Morphology of MCF7 treated with cobalt complexes, urea and doxorubicin. (**A**) Control; (**B**) Dox (50 nM); (**C**) 32 µM (**III**); (**D**) 32 µM (**IV**); (**E**) 32 µM (**III**)+2mM urea; (**F**) 32 µM (**IV**) + 2 mM urea; (**G**) 32 µM (**III**) + 2 mM urea + 50 nM Dox; (**H**) 32 µM (**IV**) + 2 mM urea + 50 nM Dox.

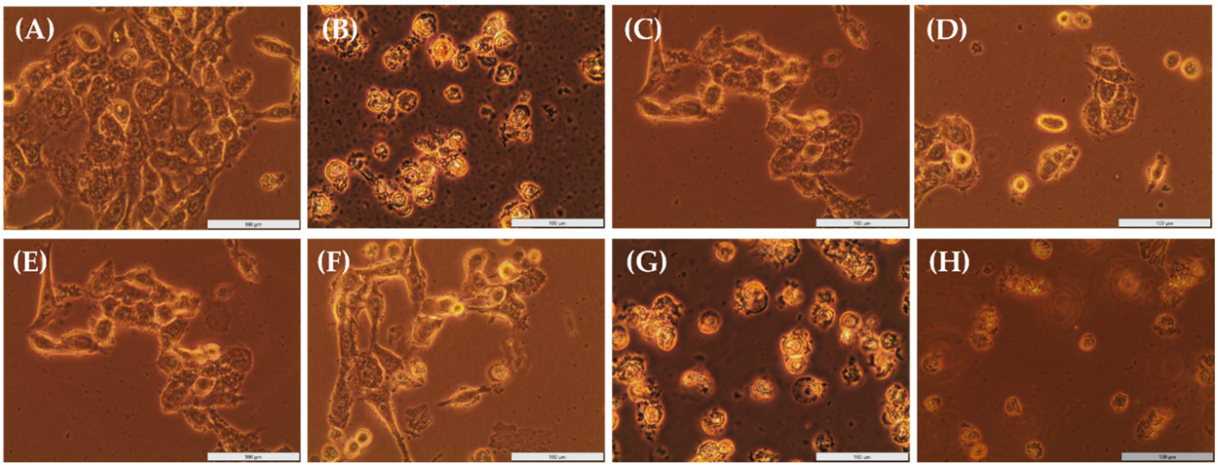

Figure S11. Morphology of HKESC-1 treated with cobalt complexes, urea and doxorubicin. (**A**) Control; (**B**) Dox (50 nM); (**C**) 32 µM (**III**); (**D**) 32 µM (**IV**); (**E**) 32 µM (**III**)+2mM urea; (**F**) 32 µM (**IV**) + 2 mM urea; (**G**) 32 µM (**III**) + 2 mM urea + 50 nM Dox; (**H**) 32 µM (**IV**) + 2 mM urea + 50 nM Dox. Signs of cell death with rounding and detachment of cells is evident in the images of treatment groups. Scale bar: 100µm.
